# Lack of racial and ethnic diversity in lung cancer cell lines contributes to lung cancer health disparities

**DOI:** 10.3389/fonc.2023.1187585

**Published:** 2023-11-01

**Authors:** Christopher Leon, Eugene Manley, Aaron M. Neely, Jonathan Castillo, Michele Ramos Correa, Diego A. Velarde, Minxiao Yang, Pablo E. Puente, Diana I. Romero, Bing Ren, Wenxuan Chai, Matthew Gladstone, Nazarius S. Lamango, Yong Huang, Ite A. Offringa

**Affiliations:** ^1^Norris Comprehensive Cancer Center, Keck School of Medicine, University of Southern California, Los Angeles, CA, United States; ^2^Department of Surgery, Keck School of Medicine, University of Southern California, Los Angeles, CA, United States; ^3^Department of Biochemistry and Molecular Medicine, Keck School of Medicine, University of Southern California, Los Angeles, CA, United States; ^4^SCHEQ Foundation, New York, NY, United States; ^5^Department of Translational Genomics, Keck School of Medicine, University of Southern California, Los Angeles, CA, United States; ^6^Hastings Center for Pulmonary Research, Keck School of Medicine, University of Southern California, Los Angeles, CA, United States; ^7^Department of Mechanical and Aerospace Engineering, University of Florida, Gainesville, FL, United States; ^8^College of Pharmacy and Pharmaceutical Sciences, Institute of Public Health, Florida A&M University, Tallahassee, FL, United States

**Keywords:** lung cancer, cell lines, underrepresented, diversity, cancer health disparities, lung adenocarcinoma, squamous cell lung cancer, *in vitro* models

## Abstract

Lung cancer is the leading cause of cancer death in the United States and worldwide, and a major source of cancer health disparities. Lung cancer cell lines provide key *in vitro* models for molecular studies of lung cancer development and progression, and for pre-clinical drug testing. To ensure health equity, it is imperative that cell lines representing different lung cancer histological types, carrying different cancer driver genes, and representing different genders, races, and ethnicities should be available. This is particularly relevant for cell lines from Black men, who experience the highest lung cancer mortality in the United States. Here, we undertook a review of the available lung cancer cell lines and their racial and ethnic origin. We noted a marked imbalance in the availability of cell lines from different races and ethnicities. Cell lines from Black patients were strongly underrepresented, and we identified no cell lines from Hispanic/Latin(x) (H/L), American Indian/American Native (AI/AN), or Native Hawaiian or other Pacific Islander (NHOPI) patients. The majority of cell lines were derived from White and Asian patients. Also missing are cell lines representing the cells-of-origin of the major lung cancer histological types, which can be used to model lung cancer development and to study the effects of environmental exposures on lung tissues. To our knowledge, the few available immortalized alveolar epithelial cell lines are all derived from White subjects, and the race and ethnicity of a handful of cell lines derived from bronchial epithelial cells are unknown. The lack of an appropriately diverse collection of lung cancer cell lines and lung cancer cell-of-origin lines severely limits racially and ethnically inclusive lung cancer research. It impedes the ability to develop inclusive models, screen comprehensively for effective compounds, pre-clinically test new drugs, and optimize precision medicine. It thereby hinders the development of therapies that can increase the survival of minority and underserved patients. The noted lack of cell lines from underrepresented groups should constitute a call to action to establish additional cell lines and ensure adequate representation of all population groups in this critical pre-clinical research resource.

## Introduction

Lung cancer remains the leading cause of cancer death in the United States (1, 2) and in the world (3) and is a prominent source of cancer health disparities (4). In the United States, Black men have the highest rate of lung cancer mortality among all groups (5). Lung cancer deaths in the United States have steadily declined due in large part to a decrease in smoking rates, particularly within Black men and women (4, 6). As a result, the gap in lung cancer deaths between Black and White men is slowly closing (1). Yet Black men in the United States still show a 12% higher lung cancer incidence rate and a 15% higher lung cancer death rate compared to White men (4, 6). Many factors are thought to contribute to this disparity, including socioeconomic factors, such as a lower frequency of screening, lack of awareness of and access to molecular testing, lack of awareness and participation in clinical trials, mistrust of the medical profession, and lack of diversity in the biomedical workforce (7–9). Importantly, genetic differences between Black and White subjects likely also play a role (10–14), with further studies required to uncover additional associations (15). It has been determined that genetics can affect lung cancer risk (16, 17), for example through differences in nicotine and carcinogen uptake (18–24) or the strength of detoxification responses (10, 25–27). Genetic background/ancestry can also affect the nature of driver mutations acquired by tumors (28–32), tumor mutational burden (33), and patient response to therapy (34). Given the numerous possible effects of genetic background on lung cancer development, pathology, and treatment, it is vital that race/ethnicity be considered in lung cancer research (35).

There are many established model systems to study lung cancer *in vitro* or *in vivo* (36). Among these, lung cancer cell lines represent a versatile and relatively affordable resource that can be widely disseminated to the scientific community (36, 37). Cell lines can be used to gain molecular insights into the development and progression of lung cancer and to pre-clinically test prospective lead candidate drugs (36, 37). Given the disproportionate impact of lung cancer on Black individuals as documented in the United States (4–6), we investigated the availability of lung cancer cell lines from Black and other underrepresented population groups, in order to determine whether available cell lines adequately represent the diversity in histological type, gender, race, and ethnicity required for optimal lung cancer research. The current review summarizes our findings.

## Lung cancer types

**Figure 1:**
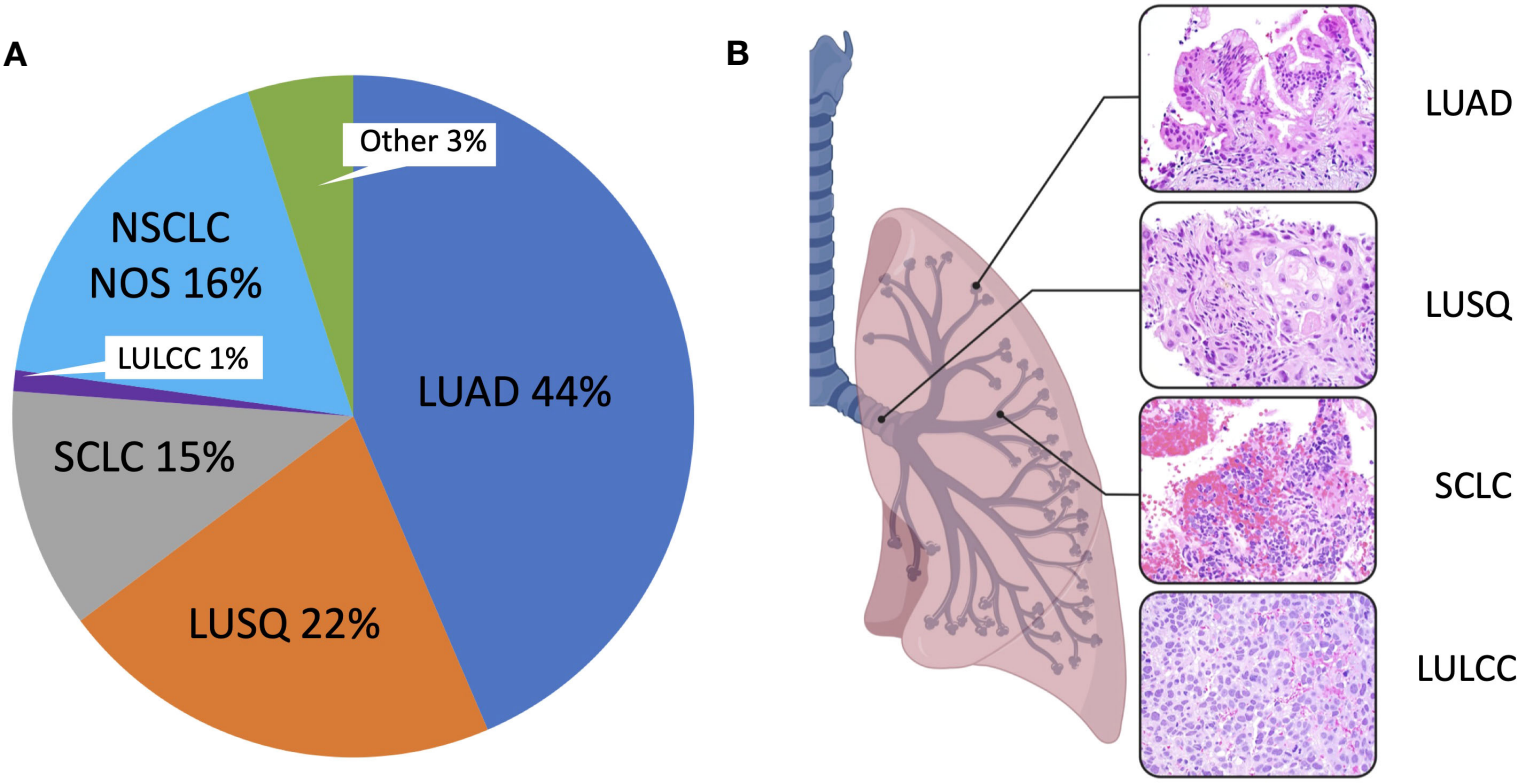
Major lung cancer histological subtypes. **(A)** Pie chart showing mortality data indicating the proportion of different histological subtypes. Mortality data was obtained from the Surveillance, Epidemiology, and End Results (SEER) Program based on 17 registries in different regions of the United States (www.seer.cancer.gov). SEER*Stat Database: Incidence-Based Mortality - SEER Research Data, 17 Registries, Nov 2021 Sub (2000-2019) - Linked To County Attributes - Time Dependent (1990-2019) Income/Rurality, 1969-2020 Counties, National Cancer Institute, DCCPS, Surveillance Research Program, released April 2022, based on the November 2021 submission. Mortality was calculated via incidence-based mortality (IBM), a method to capture population-level mortality which can be attributable to particular tumor types or other variable reported to SEER registries. IBM calculations were done as described (38). ICD-O-3 morphology codes were grouped together to form the main histologic subtypes, as described (39). LUAD, Lung adenocarcinoma; LULCC, Lung large cell carcinoma; LUSQ, Lung squamous cell cancer; NOS, Lung cancer, not otherwise specified; Other, Other specified carcinoma, including but not limited to carcinoid carcinoma, adenosquamous carcinoma, salivary gland-type carcinomas; SCLC, Small cell lung cancer. **(B)** Hematoxylin and eosin-stained sections of different lung cancer types at 400x magnification.

An important consideration for the use of cancer cell lines is that they must represent the diversity of cancer types for a given organ. In the case of lung cancer, the major histological types of lung cancer should be represented. Historically, four major histological types were designated: lung adenocarcinoma (LUAD), squamous cell carcinoma (LUSQ), small cell lung cancer (SCLC), and large cell carcinoma (LULCC) (**Figure 1**). Based on the 2015 World Health Organization reclassification of the 2004-designated lung cancer histological types, these four groups were reclassified into three major types: Lung adenocarcinoma, squamous cell carcinoma, and neuroendocrine tumors (40–42), the latter including small cell lung cancer and large cell carcinoma. Lung adenocarcinoma (LUAD), arising in the air sacs (alveoli) of the distal lung, is the most frequently occurring histological type and commonly presents in the following subtypes: lepidic, acinar, papillary, micropapillary, and solid (42). In addition, LUAD can present as invasive mucinous, colloid, fetal, enteric, and minimally invasive (42). Squamous cell lung cancer (LUSQ) is thought to arise in the airways, is the second most common major lung cancer type, and shows clearly present squamous morphologic patterns. LUSQ can be subclassified as keratinizing, nonkeratinizing, and basaloid. Within neuroendocrine tumors, the most common type is small cell lung cancer (SCLC), a very aggressive cancer that is thought to arise mainly from rare pulmonary neuroendocrine cells [though therapy-resistant lung adenocarcinoma can recur as SCLC, through genetic alterations and a possible stem cell intermediate (43, 44)]. Large cell lung carcinomas (LULCC) are poorly differentiated and when neuroendocrine morphology or staining patterns are seen, large cell lung cancers are referred to as large cell neuroendocrine carcinomas. Large cell carcinomas lacking neuroendocrine markers have been largely reclassified and assigned to other groups depending on immunohistochemical analyses, leaving only a small group of highly undifferentiated cancers designated as large cell carcinomas (~ 1%) (42).

**Figure 2:**
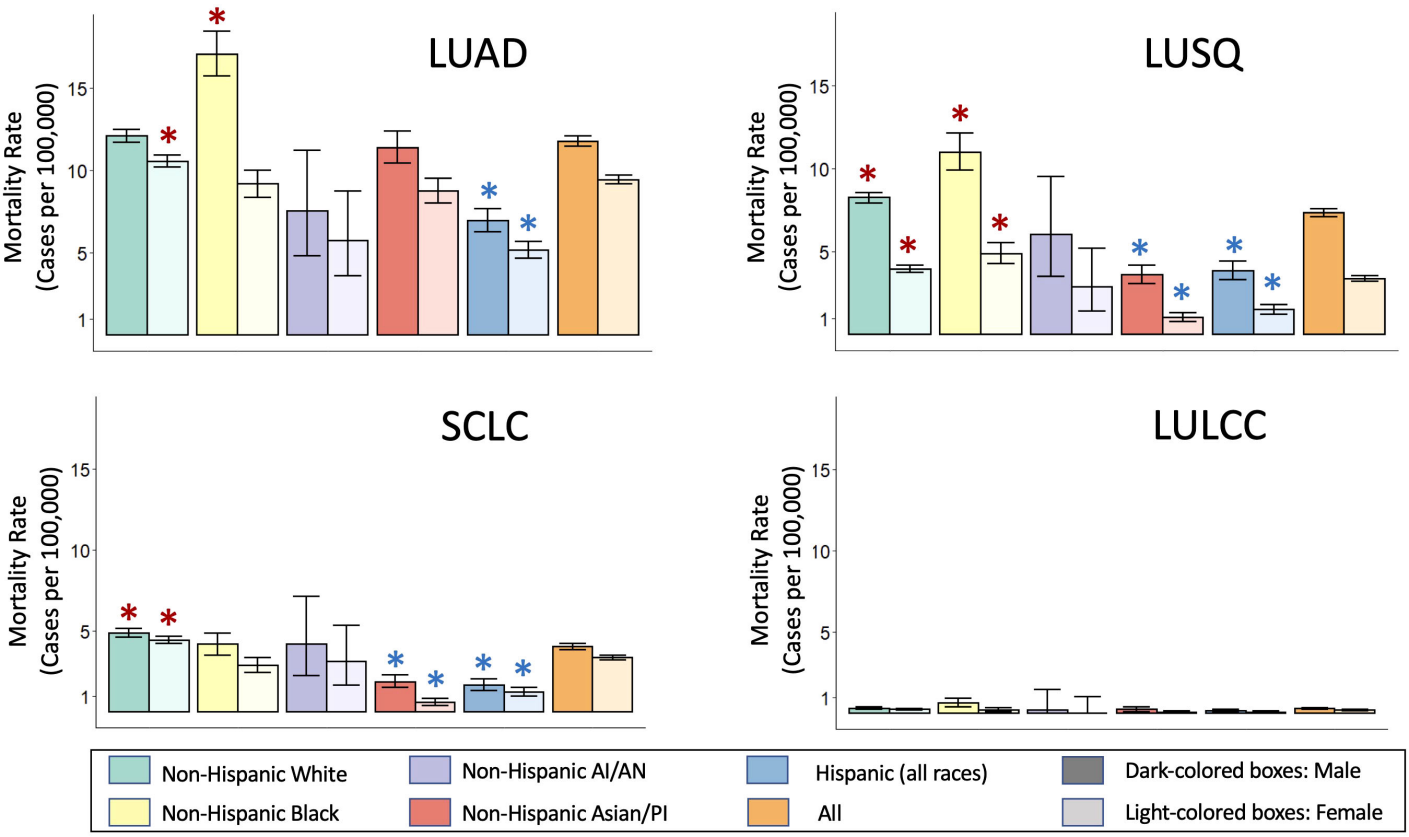
Race and ethnicity-specific mortality data for lung cancer histological subtypes. Mortality data was obtained from the Surveillance, Epidemiology, and End Results (SEER) Program based on 17 registries in different regions of the United States (www.seer.cancer.gov). SEER*Stat Database: Incidence-Based Mortality - SEER Research Data, 17 Registries, Nov 2021 Sub (2000-2019) - Linked To County Attributes - Time Dependent (1990-2019) Income/Rurality, 1969-2020 Counties, National Cancer Institute, DCCPS, Surveillance Research Program, released April 2022, based on the November 2021 submission. Mortality rates are given for each group per 100,000 individuals in that group. Mortality rates were calculated via incidence-based mortality (IBM), a method to capture population-level mortality which can be attributable to particular tumor types or other variable reported to SEER registries. IBM calculations were done as described (38). ICD-O-3 morphology codes were grouped together to form the main histologic subtypes, as described (39). Rate ratio comparison for mortality between individual race/ethnic groups compared to the overall rate of the respective gender were requested as outputs from SEER*Stats, which utilizes the Tiwari method (45). Taking into account a Bonferroni correction for the 40 comparisons, made, we considered the rate ratio significantly different compared to the “All” rate of their respective gender (orange bars) for p-value<0.00125. *,Significantly higher than the All rates for that gender; *,Significantly lower that the All rates for that gender. LUAD, Lung adenocarcinoma; LULCC, Lung large cell carcinoma; LUSQ, Lung squamous cell cancer; SCLC, Small cell lung cancer.

We used data from the Surveillance, Epidemiology and End Results (SEER, https://seer.cancer.gov/) program, a large United States-based cancer registry that at present includes over 331 million subjects from 17 regions, to assess lung cancer mortality for different races/ethnicities for the main histological types (**Figure 2**). The data shows LUAD as the most common histological type across all gender and racial/ethnic categories. Black men and White women show the highest age-adjusted mortality rates for LUAD. Squamous cell lung cancer is the second most common histological type, with Black men and women showing the highest age-adjusted mortality rates for LUSQ. Age-adjusted mortality rates for SCLC are highest for White men and women, while for LULCC they are highest for Black men. Cell lines have been established from the most common lung cancer types (36), with lung adenocarcinoma cell lines predominating because cultures were relatively easy to establish. It should be noted that the histological classifications of cell lines are based on the WHO classification in use at the time the lines were established and may thus not fully match current designations.

## Cell lines as model systems

Lung cancer cell lines allow the *in vitro* study of human lung cancer, and are especially important for facilitating research when tumor samples are difficult to obtain. SCLC is one such case; it is rarely surgically resected because patients usually present with metastases. In principle, cell lines can be propagated indefinitely and relatively cheaply and are easily disseminated, which allows different labs to study the same cells and compare their results, for example in drug screens. Another advantage of cancer cell lines is that they are pure populations of cells, lacking contaminating stroma and other cell types, thereby allowing detailed genetic and epigenetic studies. This lack of context also has its drawbacks, but these can be addressed using certain culture conditions and model systems as described in a later section.

The first cancer cell line to be cultured was the HeLa cell line, derived from Henrietta Lacks, a black woman with cervical cancer (46). Important ethical questions have been raised about the fact that the cells were obtained at the time without informed consent from the patient (47). The establishment of the HeLa cell line was a scientific breakthrough, and HeLa cells have been widely used in academic and biotech laboratories (47). The demonstrated ability to culture tumor cells from a human patient set the stage for subsequent work establishing cell lines from many kinds of cancer, including lung cancer cell lines.

Due in large part to intensive efforts by Drs. Gazdar, Minna, and Carney to optimize methods to derive cell cultures from patient lung tumors, a large number of cell lines were established (37, 48). With their collaborators, these investigators ultimately cultured more than 200 lung cancer cell lines of different histological types, initially at the National Cancer Institute (NCI-designated lung cancer cell lines), and later at UT Southwestern Medical Center at the Hamon Cancer Center (HCC-designated cell lines). Combined with the efforts of other investigators across the world, over 400 lung cancer cell lines have been reported (36). Many of these cell lines have been cultured for decades, raising concerns among some investigators that the lines might experience genetic drift. Fortunately, the genetic and epigenetic alterations seen in lung cancer cell lines have remained relatively stable over time (37). It has also been asked how well the obtained cell lines represent the tumors from which they are derived. A comparison between a large number of lung cancer cell lines and primary lung cancers has demonstrated that many key genetic and epigenetic changes seen in lung cancer tumors have also been observed in cell lines (37).

## Cell line quality and authentication

Two important considerations when using cell lines for research are cell line quality and authenticity. The presence of contaminating microorganisms, particularly mycoplasma, and the cross-contamination with other cell lines can invalidate performed research (49, 50). Mycoplasma is a type of infectious prokaryote lacking a rigid cell wall. While primary cells can be contaminated, laboratory personnel can also be a source of infection (49). Infection can affect cell growth and physiology, thereby nullifying experimental results and making it imperative that cultures be routinely tested so that contaminated cultures be discarded (49, 51–54). If discarding is not an option because a cell line is rare or even irreplaceable, treatment with antibiotics may be considered (55). Authentication of cell lines is also of critical importance. A previous lack of cell line authentication has resulted in large numbers of publications based on incorrect cell types, including many cell lines found to be, in truth, HeLa cells (56). Thus, cell lines should be obtained from reliable sources and should be routinely authenticated through DNA fingerprinting, i.e., the use of short tandem repeats (STRs) (47, 51, 53, 54, 57) (see **Table 1** for useful web sites). Journals and granting agencies can help minimize misidentification by requiring authors to authenticate cell lines used in publications (58, 59), such as required by the National Institutes of Health (https://grants.nih.gov/grants/guide/notice-files/not-od-15-103.html). Current recommendations are to test cell lines for mycoplasma and authenticity when they first reach a new laboratory, before publication, and every two months while in culture.

**Table 1:** Cell line verification web sites.

Goal	Web site
Identify cell lines	https://www.atcc.org/search-str-database
Identify cell lines	https://www.cellosaurus.org/
Identify cell lines	https://www.dsmz.de/services/human-and-animal-cell-lines/online-str-analysis
Find mislabeled cell lines	https://www.atcc.org/the-science/authentication/reclassified-cell-lines

## Representation of different races/ethnicities in current lung cancer cell line collections

We investigated the availability of lung cancer cell lines representing different races/ethnicities using the resources listed in **Table 2**. We identified over 800 lung cancer cell lines (**Supplementary Table 1**). A substantial fraction of these are isogenic (derived from the same parental line) or derived from different sites of the same patient. We identified almost 200 lung cancer cell lines from White subjects (**Supplementary Table 1A**), 6.5-fold more than the 31 cell lines available from Black patients (**Table 3**, **Supplementary Table 1B**). One of the 31 cell lines appears to be a duplicate (NCI-H2108 lists identical patient age, gender, cancer histology, and cell line STR analysis to NCI-H2107). Of the 30 unique Black lung cancer cell lines, 6 were derived from lung adenocarcinomas, 4 from squamous cell cancers, 11 from SCLCs, and the remainder were from unspecified non-small cell lung cancers (3), adenosquamous carcinomas (2), large cell carcinomas (2), a carcinoid tumor, a giant cell carcinoma, and a mucinoepidermoid carcinoma. Ancestry information was available for 24 of these lines, and showed African ancestry, ranging from 56% to 91%.

**Table 2:** Resources from which lung cancer cell line information was obtained.

Resource Name	Web site
ATCC: The Global Bioresource Center	https://www.atcc.org/
cBioPortal for Cancer Genomics	https://www.cbioportal.org/
Expasy - Cellosaurus.	https://www.cellosaurus.org/
Wellcome Sanger Institute. Cell model Passports. A Hub for Preclinical Cancer Models.	https://cellmodelpassports.sanger.ac.uk/passports?tissue=lung

Additional data was obtained from the literature (36, 37, 50, 60–69).

**Table 3:** Lung cancer cell lines from Black patients.

Name	Sex	Age	Histol. Type	Smoking	% African	Mutations
201T	M	68Y	LUAD	U	89	*TP53*
HCC1195	M	47Y	LUAD	U	70	*TP53, NRAS*
HCC122	M	48Y	LUAD	U	U	U
NCI-H23	M	51Y	LUAD	U	68	*TP53, KRAS, STK11, ATM*
NCI-H1373	M	56Y	LUAD	SM (30 py)	72	*TP53, KRAS*
NCI-H1648	M	39Y	LUAD	SM	69	*TP53*
NCI-H125*	M	61Y	LUADSQ	U	U	*TP53*
NCI-H513	M	61Y	LUADSQ	U	84	U
HLF-a**	F	54Y	LUSQ	U	91	U
NCI-H1385	F	49Y	LUSQ	SM (33 py)	69	*KRAS*
HCC15	M	47Y	LUSQ	U	77	*TP53, RB1, NRAS, EP300, CTNNB1*
HCC1897	M	47Y	LUSQ	U	77	U
NCI-H64	F	48Y	SCLC	SM (30 py)	68	*TP53*
NCI-H128	M	60Y	SCLC	U	70	*TP53*
NCI-H220	M	51Y	SCLC	NS	U	U
NCI-H250	M	34Y	SCLC	NS	91	*TP53, RB1*
NCI-N390	M	49Y	SCLC	U	U	U
NCI-H748	M	62Y	SCLC	SM (30 py)	86	*TP53, BRCA2*
NCI-H1048	F	53Y	SCLC	NS	70	*TP53, RB1, PIK3CA*
NCI-H1339	F	49Y	SCLC	U	71	*TP53*
NCI-H1963	M	56Y	SCLC	U	56	*TP53, RB1*
NCI-H2107	M	36Y	SCLC	U	U	*TP53*
NCI-H2108***	M	36Y	SCLC	SM (26 py)	U	U
NCI-H835	F	48Y	LUCART	NS	80	U
HCC1359	F	55Y	LUGCC	U	86	*TP53*
HCC3051	M	63Y	LULCC	U	U	U
NCI-H810	M	51Y	LuLCC	U	82	*TP53, DDR2*
NCI-H292	F	32Y	LUMEC	U	81	*NF2*
EMC-BAC-1	M	U	NSCLC	U	74	*TP53, STK11*
NCI-H2110	U	U	NSCLC	NS	83	U
NCI-H2172	F	U	NSCLC	NS	82	U

*Cell line discontinued; **Cell line reported to be contaminated; ***Duplicate cell line (H2107); Sex: M, Male; F, Female; Age: Y,Years; Subtype: LUAD, Lung adenocarcinoma; LUADSQ, Lung adenosquamous carcinoma; LUCART, Lung carcinoid tumor; LUGCC, Lung giant cell carcinoma; LULCC, Lung large cell carcinoma; LUMEC, Lung mucoepidermoid carcinoma; LUSQ, Lung squamous cell cancer; NSCLC, non-small cell lung cancer; SCLC, Small cell lung cancer; Smoking: NS, non-smoker; SM, Smoker; py, pack years; U, Unknown; % African: Percentage African ancestry; U, Unknown; Mutations: known mutations are indicated; U, Unknown. Additional information can be found in **Supplementary Table 1B**.

We identified 390 cell lines from Asian lung cancer patients (**Supplementary Table 1C**), of which 20% appear to be non-unique (e.g. from different metastatic sites in the body of a given patient), or sister cell lines derived through manipulation of the original cell line.

We did not identify any cell lines representing H/L, AI/AN, or NHOPI individuals. It is possible that H/L ethnicity has not been properly documented for existing cell lines and thus, that such cell lines might be present in the current collection. However, while cell line race can be retrospectively examined using ancestry informative markers (single nucleotide polymorphisms that help infer ancestry admixtures (70–73)), H/L individuals in the Unites States represent an admixture population that may include White, Black, and AI/AN components and would be difficult to genetically identify. Going forward, ethnicity information would need to be documented at the time of sample collection.

We found almost 300 cell lines for which race/ethnicity is unknown (**Supplementary Table 1D**). Thus, there may be Black, AI/AN, and NHOPI cell lines among these unclassified lines and it may be worth determining their genetic ancestry (70–73).

We noted that the number of cell lines developed from men was over 2-fold higher than cell lines developed from women, and this excess was most prominent for the cell lines developed from Asian individuals (almost 7-fold) (**Supplementary Table 1**). This difference exceeds what might be expected based on the higher frequency of lung cancer detected in males, and indicates a disparity in the representation of female individuals in lung cancer cell lines. Overall, we conclude that there is a marked lack of cell lines from underrepresented populations and an underrepresentation of cell lines from women.

## Cell lines representing the cells-of-origin of different types of lung cancer

In addition to cell lines derived from tumors, it is also important to establish cell lines derived from the cells-of-origin for the different lung cancer histological types. These cells can be useful for modeling the sequential development of the different lung cancer histological types and the effects of environmental exposures on lung cells from the airway or alveolar compartments. Genetic background can affect lung cancer predisposition as well as the metabolism and detoxification of tobacco smoke components (10, 18–27, 74). Thus, just as we need lung cancer cell lines from different races and ethnicities, we need cell-of-origin cell lines from different races and ethnicities to appropriately model lung cancer development. Normal lung cells derived from humans are not immortal and will undergo senescence when propagated *in vitro* (75). Immortalized cell lines must therefore be created using either viral genes such as Simian Virus 40 large T antigen (SV40LgT) (76, 77) or human papillomavirus E6+E7 genes (78), or overexpression/modification of human genes that allow cell cycle progression and prevent telomere shortening and the resulting senescence (79).

LUAD arises from alveolar epithelium, and to model human lung adenocarcinoma development *in vitro*, human immortalized alveolar epithelial cells are required. Four immortalized alveolar epithelial cell lines (hAECs) were established using SV40LgT antigen (80, 81). Race is only known for 3 of these cell lines, which were derived from White subjects (81). In addition, a polyclonal alveolar epithelial cell line of unknown race/ethnicity was established using a proprietary cocktail of 33 immortalization genes (82) and from it, a monoclonal cell line (Arlo) was recently derived (83). It will be important to develop additional immortalized alveolar epithelial cell lines for other racial/ethnic groups, given that LUAD is the most common lung cancer histological type in the United States for both genders and all races and ethnicities (**Figure 2**).

Human bronchial epithelial cells, the putative cells of origin of LUSQ, have been immortalized with SV40LgT, resulting in the BEAS-2B cell line (84), and by using overexpression of the telomerase gene in combination with either overexpression of G1 cell cycle kinase CDK4 or short hairpin RNA-based knockdown of cell cycle regulatory proteins p16^INK4A^ and p14^ARF^. The latter yielded human bronchial epithelial cells (HBECs) and small airway epithelial cells (SAECs) (85, 86). BEAS-2B, HBEC, and SAEC cell lines can be useful to model the development of squamous cell lung cancer or determine the effects of environmental exposures on airway cells. To our knowledge, the race/ethnicity of the individuals from whom the cell lines were derived is unknown. Thus, ancestry tests of these lines would be useful, as would establishing more of these types of cell lines representing diverse races.

The availability of methods to establish immortalized alveolar and airway cells allows progress to be made in deriving additional cell lines from racially and ethnically diverse subjects. However, there is one important cell type for which no immortalized human cell lines have yet been established: pulmonary neuroendocrine (PNE) cells, the main cell-of-origin of SCLC (87). Immortalized PNE cells would be an important added tool to study the development of SCLC and may be especially relevant for studies of Black SCLC, as this type of cancer may arise at an earlier age in Black subjects than in other races (88, 89). However, PNE cells are rare (less than 1% of lung epithelial cells) making their isolation and immortalization challenging. One possible strategy is to derive these cells from induced pluripotent stem cells (iPSCs), a feat that was recently achieved (90).

Derivation of cell line types from induced pluripotent cells has also been used to obtain bronchial epithelial cells (91) and alveolar epithelial cells (92–94). The availability of racially/ethnically diverse iPSCs (95) provides an opportunity to derive diverse cell lines representing lung cancer cells-of-origin. However, iPSC-derived cell populations can consist of mixed cell types, and considerable time and expertise are required to differentiate them correctly (96, 97). Whether the epigenomes of such iPSC-derived cells fully match those of the corresponding adult differentiated cell types would also need to be determined. Using cell lines with the correct initial epigenome is particularly relevant in studies of the effect of environmental exposures (98). Epigenetic changes play a role in the development of all cancer types (99) and can be driven by environmental exposures such as tobacco smoke (100, 101). Using cell lines with epigenomes matching the natural cells-of-origin is also highly relevant for the study of disease-risk single nucleotide polymorphisms (SNPs) (98). Most risk SNPs, including those for lung cancer, lie in intergenic regions or introns, and likely affect risk by introducing changes in epigenetic regulatory elements (102). If cells differentiated from iPSCs do not epigenetically match their normal mature counterparts, regulatory elements may be missing or altered, thus affecting the correct interpretation of risk SNP epigenetic environments.

## Applications of lung cancer cell lines and immortalized lung cell lines

Lung cancer cell lines and cell-of-origin cell lines can be used in a wide variety of ways to study lung cancer (36). In the simplest form, they can be grown on Petri dishes in two-dimensional culture or, in the case of classic SCLC cell lines, in suspension (48). Such *in vitro* cultures can be useful for the study of cancer driver and tumor suppressor genes, epigenetic changes in cancer cells, the effects of environmental exposures, and the investigation of lung cancer risk SNPs, among other topics. Cell lines provide a relatively pure population of cells compared to heterogeneous tumor or tissue samples that can contain variable amounts of contaminating blood cells and stroma. This simplification can greatly facilitate analyses and provides one powerful strategy to leverage cell-based models. However, it lacks the complexity arising from growth in three-dimensional space or from the interactions with other cell types, such as fibroblasts and blood vessels. Growth of pure cell lines with a defined medium in three dimensions can provide the next level of complexity, while the addition of fibroblasts, endothelial, and blood cells can further simulate *in vivo* characteristics. Even further advanced are three-dimensional models, so-called “organs-on-a-chip”, which may incorporate an air-liquid interface and/or the movement associated with breathing (103, 104). Organ-on-a-chip devices allow epithelial cells to be coated on a main channel and supportive cells or endothelial cells on a parallel secondary channel separated by a thin porous membrane (105). They can be used to study cancerous cells or cancer cells-of-origin, and should be considered for drug testing as the cellular microenvironment can affect cancer cells’ susceptibility to drugs (106, 107).

No matter how advanced an *in vitro* model is, it will not provide a natural tumor microenvironment identical to that found *in vivo*. To achieve the latter, implantation of cell lines into model organisms such as mice is required. To avoid rejection, immunocompromised (“nude”) mice or humanized mice need to be used. Such models, known as xenografts, can be made using human cancer cell lines, primary patient tumors, or even circulating tumor cells (108). Subcutaneous implantation is often used; while not fully mimicking the natural microenvironment, it allows easy monitoring of tumor size and thereby any therapeutic responses. However, if the cells used do not capture the racial and ethnic diversity of lung cancer patients, all models will fall short in moving lung cancer research forward for all population groups.

## Discussion

Lung cancer cell lines and cell lines from lung cancer cells-of-origin are a key part of the research toolkit needed to advance knowledge on the development, progression, diagnosis, and treatment of lung cancer. However, in order to ensure that the knowledge gained, tools developed, and treatments devised are applicable to the population regardless of race or ethnicity, we need to ensure that cell lines representing all groups are available. In particular, cell lines representative of Black males should be at hand as Black males show the highest rates of lung cancer death. Here, we investigated the availability of lung cancer cell lines from underrepresented minority populations. We identified over 800 lung cancer cell lines, including ~200 unique lung cancer cell lines from White subjects and over 300 from Asian subjects. This contrasted with just 30 unique lung cancer cell lines available from Black patients. No lung cancer cell lines from H/L, AI/AN, or NHOPI individuals were identified, though some maybe present among the almost 300 lung cancer cell lines of unknown race/ethnicity. It is important to carry out ancestry analyses of existing cell lines to verify which race these lines best represent. In addition, a concerted effort should be made to generate more cell lines from women and underrepresented groups, and to document ethnicity at the time of tissue collection. Expanding the cell line repertoire is even more relevant for cell-of-origin lines, of which there are very few, and to our knowledge none from underrepresented groups.

It should be considered that certain racial/ethnic groups may have cultural objections to donating cells or tissues. Those desires should be respected, even if it means that population groups may not be represented in research. It is also important to keep in mind that broadly defined race/ethnicity groups do not capture the heterogenicity of admixed populations. For example, an analysis of Hispanic men in Florida showed that while lung cancer mortality rates were lower than those of White men, they were 50% higher in Puerto Rican than non-Puerto Rican men (109). Once cell lines from all groups willing to participate have been collected and represent all three major lung cancer types and cells-of-origin (from both men and women), thought should be given to key subpopulations that may merit disaggregation.

One short-term way to partially alleviate the current paucity of lung cancer cell lines representing different racial/ethnic groups is to use genome engineering to derive isogenic cell lines from the handful of underrepresented cell lines available. Cancer driver genes present in the cell lines can be replaced by other driver genes to generate cell lines in which the effects of different driver genes within a similar genomic context can be examined. This would expand the cell line repertoire available for molecular and drug development studies. However, to do this in a biologically meaningful way, the key cancer driver genes present in the different racial/ethnic populations of lung cancer patients must be identified. Unfortunately, cancer driver genes in underrepresented populations are under-studied. For example, in the public database The Cancer Genome Atlas (https://www.cancer.gov/ccg/research/genome-sequencing/tcga), the number of sequenced lung cancer samples from White patients outnumbers that of Black patients by almost 9:1. Thus, data on driver mutations in underrepresented patients must also be expanded. Clearly, much work remains to be done. The first step is to highlight current shortcomings in knowledge and resources, and to disseminate information to lung cancer patients of all races and ethnicities about the need for cell lines representing lung cancer in their communities. Explaining how lung cancer cell lines and cell-of-origin lines can be used to improve research and develop new therapies for people of the patients’ own racial/ethnic backgrounds can help patients make an informed decision about whether to participate. In addition, it would be beneficial if the donations of tissues/cells were discussed with patients by researchers and/or clinicians from their own racial/ethnic group, supporting mutual trust and a better understanding of research goals (7–9). To this end, all races and ethnicities should be well-represented in the medical and biomedical research professions. Thus, we need to build not only the tools, but also foster the success of clinicians and biomedical researchers who can advocate for the establishment and implementation of those tools.

## Author contributions

CL, EM, DIR, NSL, PEP, YH and IAO conceived of the study. CL identified and tabulated lung cancer cell lines. CL, AMN, MRC, DAV, MY, BR, WC, DIR, and MG drafted sections of the manuscript. JC extracted data from SEER and generated figures, all authors assisted with editing, and IAO oversaw and finalized the manuscript. All authors contributed to the article and approved the submitted version.

## Positionality statement

The authors represent the following racial/ethnic groups: White Hispanic/LaIn(x) (CL, JC, MRC, DAV, PEP, DIR, MG), Black non-Hispanic(EM,Jr, AMN, NSL), White non-Hispanic (IAO), and Asian (MY, BR, WC, YH).
